# Alkalinity enrichment stimulates calcification and linear extension in *Acropora cervicornis*

**DOI:** 10.1038/s41598-026-44817-6

**Published:** 2026-03-22

**Authors:** Kenzie M. Cooke, Ana M. Palacio-Castro, Albert Boyd, Nash Soderberg, Patrick M. Kiel, Ashley Stevens, Chris Langdon, Ian C. Enochs

**Affiliations:** 1 Cooperative Institute for Marine and Atmospheric Studies, Miami, FL USA; 2 Rosenstiel School of Marine, Atmospheric, and Earth Science, University of Miami, Miami, FL USA; 3 Atlantic Oceanographic and Meteorological Laboratory, Miami, FL USA

**Keywords:** Coral restoration, Coral husbandry, Aquaculture, Coral growth, Alkalinity enhancement, Skeletogenesis, Ecology, Ecology, Environmental sciences, Ocean sciences

## Abstract

Environmental change, disease, and a myriad of local stressors have led to worldwide declines in coral cover, demanding that restoration efforts scale with the magnitude of the crisis. Critical to this goal is the industrial-scale propagation of coral fragments. Preliminary evidence from aquarists suggests that elevating seawater alkalinity increases coral growth, and several papers have reported enhanced calcium carbonate deposition. Many questions remain, however, concerning the effectiveness on species targeted for restoration, optimal alkalinity range, and the manner in which growth is affected (e.g., skeleton extension versus density). Here, we investigate the effect of elevated alkalinity on total calcification and linear extension of the Caribbean coral, *Acropora cervicornis*. Corals were exposed to one of four alkalinity treatments for 33 days using a sodium bicarbonate/sodium carbonate solution. Elevated alkalinity significantly enhanced total calcification, increasing by 125% in the highest treatment. Linear extension was also significantly enhanced during the first half of the experiment (98%), but the effect was no longer detectable after week three when growth slowed in all groups, including the controls. These findings suggest that elevated alkalinity may accelerate coral propagation, representing an affordable and practical tool to enhance production efficiency in land-based coral restoration facilities.

**Supplementary Information:**

The online version contains supplementary material available at 10.1038/s41598-026-44817-6.

## Introduction

Over the past half century, chronic exposure to a multitude of environmental and anthropogenic stressors has led to severe declines in the health and structure of tropical coral reef ecosystems around the world^1–5^. In the Western Atlantic and Caribbean, coral cover has decreased by as much as 80%^6,7^, representing tens of thousands of impacted hectares. Rising ocean temperatures^8,9^, the resultant coral bleaching^10^, and the spread of novel disease^11^ have fundamentally restructured coral communities in the area, and the increasing frequency of these mass mortality events make natural recovery improbable^12,13^.

These challenges have spurred massive restoration efforts, which aim to restore degraded reefs by transplanting healthy coral fragments typically grown in offshore or land-based nurseries^14,15^. Despite significant advances in propagation and outplanting techniques over the past several decades^16–20^, it remains to be seen whether these “coral gardening” efforts can achieve the scale necessary to generate sustained, ecosystem-wide impacts and maintain the essential ecological services that support marine life and coastal communities^15,21,22^.

*Acropora cervicornis*, a key reef builder in Florida and the Caribbean, was the first observed taxon to suffer sharp declines beginning in the 1980s and has thus become a major focus of restoration efforts in the region^23,24^. Its fast growth rates and propensity for natural asexual reproduction relative to other local species make it an ideal candidate for large-scale propagation^25,26^. As such, *A. cervicornis* has been widely cultivated in the area but suffered significant losses across both wild populations and field-based nurseries during the 2023 marine heatwave^27–29^. These losses have stimulated a greater interest in land-based coral nurseries, in part because land-based propagation can safeguard the genetic diversity of restoration stock by limiting exposure to environmental disturbance. Ex-situ coral propagation also provides the opportunity to optimize environmental conditions and husbandry practices to promote growth and resilience (e.g., supplemented feeding^30,31^ and variable temperature regimes^32^). In addition, seawater chemistry can be carefully monitored and adjusted. Alkalinity is one such parameter that is routinely managed in saltwater aquaria and has the potential to increase coral growth when elevated beyond typical environmental levels^33–35^.

Alkalinity is essentially a measure of excess bases (proton acceptors) over acids, and reflects a solution’s capacity to resist changes in pH. In seawater, alkalinity is dominated by bicarbonate (HCO₃⁻) and carbonate (CO_3_^2−^) ions, major components of the carbonate system. Elevating alkalinity through the addition of a base (i.e., NaOH, NaHCO_3_, or Na_2_CO_3_) raises the pH and shifts the carbonate system equilibrium towards CO_3_^2−^. This subsequent increase in CO_3_^2−^ availability elevates the aragonite saturation state (Ω_Arg_), which describes the thermodynamic potential for aragonite, a crystalline form of calcium carbonate produced by corals, to precipitate, and is calculated as the product of the calcium and carbonate ion concentrations divided by the stoichiometric solubility product (K'_sp_, Eq. (1)).

1 $$\Omega = \frac{[Ca^{2+}][CO_3^{2-}]}{K_{sp}^{\prime}}$$

Because calcification is fundamentally linked to carbonate chemistry, increasing alkalinity can favor aragonite formation, with corals responding either directly to the increase in CO_3_^2−^ and HCO_3_^–^, or to associated changes in pH or Ω_Arg_^34,35^. For example, the addition of NaHCO_3_ and Na_2_CO_3_ continued to enhance calcification in an Indo-Pacific *Acropora sp.* with no observed plateau at HCO_3_^–^ concentrations exceeding four times ambient seawater (~ 7100 µM)^34^. This approach has also been shown to temporarily increase net community calcification on a natural reef flat by an estimated 7%^35^.

While most studies on alkalinity-enhanced coral growth report calcification as a single metric, it is in fact a composite process that encompasses multiple skeletal features, including densification, lateral thickening, and linear extension^36,37^. Differential impacts on any of these attributes could have physiological and ecological ramifications that would carry important implications for coral resilience^38,39^. Increased linear extension (LE), for example, may accelerate biomass production, but if it came at the expense of decreased skeletal density, it could undermine resilience to storms, predation, and ocean acidification^26,40^. While elevated HCO_3_^–^ concentrations have been shown to stimulate both skeletal densification and LE in the Indo-Pacific *Porites compressa*^33^, the relationship has yet to be verified for Caribbean species and is poorly understood in the context of restoration.

Further, the relationship between seawater chemistry and LE remains unclear, and various ocean acidification experiments have produced conflicting results. Jokiel et al.^41^, for example, found LE to be impaired under elevated *p*CO_2_, yet several studies investigating the impact of ocean acidification on growth in *A. cervicornis* report decreased total calcification, but no effect on LE^42,43^. Additionally, it has been shown that a low Ω_Arg_ does not always lead to decreased calcification^44^, and one study found that corals living along a natural pH gradient near a CO_2_ vent decreased their skeletal porosity in response to lowered pH, but maintained constant LE^45^. Conversely, *Porites* cores from seagrass meadows experiencing diel pH elevations of up to 8.5 exhibited reduced LE but increased skeletal density, resulting in greater total calcification relative to nearby reef-flat conspecifics^46^. However, across a broader inshore–offshore gradient, the seagrass-associated corals had less dense skeletons, highlighting the difficulty of isolating carbonate-chemistry effects on skeletal architecture from co-varying environmental drivers such as light, depth, and nighttime hypoxia and acidification in shallow environments. Together, these findings suggest that carbonate chemistry can differentially influence skeletal deposition, but responses are highly context-dependent, and the underlying mechanism remains unresolved.

Elevating seawater alkalinity in ex-situ facilities has the potential to promote faster growth rates and amplify restoration efforts by accelerating colony propagation, increasing biomass production, reducing early juvenile mortality^47^, and potentially shortening the time to sexual maturity of coral recruits^48^. Despite its widespread use in hobbyist aquariums and within commercial coral aquaculture, relatively few scientific studies have verified the efficacy of alkalinity manipulation. Many questions remain regarding its effectiveness on species targeted for restoration, optimal alkalinity range, and the manner in which growth is altered. Moreover, understanding how elevated alkalinity influences calcification has broader implications for predicting ecological responses to Ocean Alkalinity Enhancement (OAE), a proposed marine carbon dioxide removal intervention which could alter reef carbonate chemistry at large spatial scales^49^, with potential consequences for reef accretion, long-term persistence, and coral restoration success.

Here, we investigate the effect of elevated alkalinity on total calcification and LE in *A. cervicornis* to assess the potential impact of this technique on restoration efforts, while addressing larger gaps in our knowledge regarding the role of carbonate chemistry in coral growth dynamics. We exposed individual coral fragments (n = 40) to one of four water treatments for 33 days using a novel, automated dosing system^50^. Each coral was maintained in an independent flow-through beaker and received either ambient seawater or seawater enriched by + 1200, + 1850, and + 2000 µmol kg^−1^ above ambient total alkalinity (A_T_).

## Results

### Carbonate chemistry

**Fig. 1:**
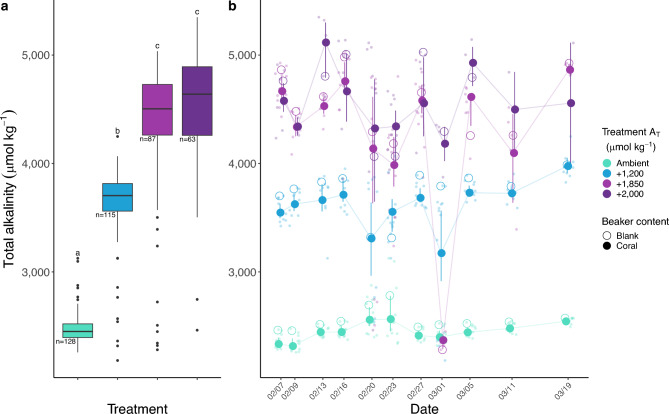
(**A**) Summary of treatment alkalinities (median ± interquartile range). The sample size used to calculate treatment summary statistics are printed below each box. (**B**) Stability of alkalinity treatments over time. Opaque circles represent discrete measurements from beakers and the dark circles represent the treatment average with standard error bars for each collection point. Open circles denote beakers containing no corals. Letters above each box indicate statistically distinct treatments; treatments that share a letter are not significantly different (Tukey’s HSD, p < 0.05).

Mean A_T_ was significantly higher in each elevated treatment compared to the ambient treatment (Tukey’s HSD: *p* < 0.0001 for all pairwise comparisons). A_T_ also increased significantly from the + 1200 to the + 1850 treatment (Tukey’s HSD: *p* < 0.0001), but not between the + 1850 and + 2000 treatments (Tukey’s HSD: *p* = 0.51; Fig. 1A). Treatment A_T_ with mean and standard error can be seen in Table 1**,** along with additional carbonate chemistry parameters generated using *seacarb*.

**Table 1:** Summary of the carbonate chemistry parameters in the experimental replicates.

Alkalinity treatment									
n	Beaker content	Ambient		+ 1,200		+ 1,850		+ 2,000	
	*Coral*	68		61		44		33	
	*Empty*	14		11		11		7	
		Mean	SEM	Mean	SEM	Mean	SEM	Mean	SEM
A_T_ (µmol kg^-1^)	*Coral*	2458.8	11.67	3670.7	37.3	4504.1	74.4	4601.4	111.1
	*Empty*	2543.5	27.2	3744.6	101.8	4540.0	92.6	4610.9	150.0
DIC (µmol kg^-1^)	*Coral*	2119.2	11.4	2983.9	28.1	3577.6	56.3	3679.0	83.2
	*Empty*	2185.4	22.9	3049.8	81.3	3581.0	72.5	3736.5	141.7
pCO_2_ (µatm)	*Coral*	418	10	324	8	368	39	441	72
	*Empty*	409	17	331	19	328	49	416	85
pH (Total)	*Coral*	8.06	0.010	8.285	0.011	8.352	0.032	8.328	0.043
	*Empty*	8.076	0.016	8.284	0.025	8.380	0.055	8.308	0.068
^Ω^Arg	*Coral*	3.98	0.08	8.76	0.20	12.28	0.61	12.34	0.88
	*Empty*	4.22	0.14	8.91	0.46	12.70	1.14	11.62	1.38
HCO^3−^ (µmol kg^−1^)	*Coral*	1860.78	12.90	2431.03	23.90	2804.07	65.24	2900.60	89.65
	*Empty*	1911.86	22.20	2487.78	69.65	2782.53	115.38	3001.41	181.54
CO_3_^2−^ (µmol kg^−1^)	*Coral*	247.13	4.80	555.09	12.15	763.61	38.16	766.46	53.29
	*Empty*	262.57	8.76	552.95	28.26	789.58	71.37	723.89	86.87
Salinity (ppt)	*Coral*	34.41	0.11	34.27	0.12	34.39	0.12	34.24	0.14
	*Empty*	34.40	0.25	34.24	0.25	34.31	0.25	34.60	0.21

Values were generated in *seacarb* using measured A_T_ and DIC. Samples with post-collection precipitation of calcium carbonate, deltas > 5 μmol, and those with *seacarb* calculated pCO_2_ > 2000 µatm were excluded. n = total number of samples from each treatment used to calculate mean and standard error values for each parameter. Values calculated separately for beakers with and without corals.

### Coral response

#### Total calcification

**Fig. 2:**
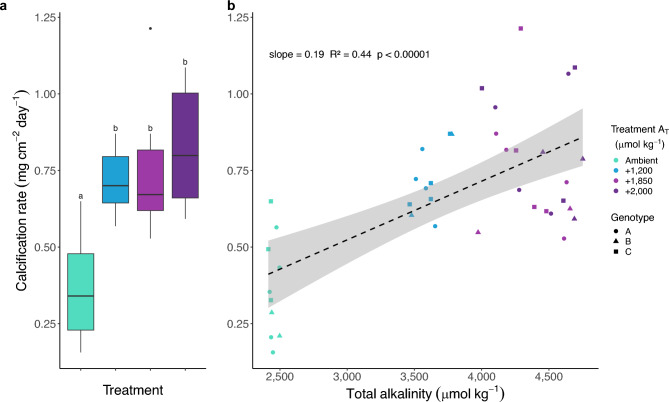
Effect of alkalinity enrichment on the calcification rate of *A. cervicornis*. (**A**) Calcification rates (median ± IQR; n = 10 per treatment). Letters above each box indicate statistically distinct treatments; treatments that share a letter are not significantly different (Tukey’s HSD, p < 0.05). (**B**) Linear regression between calcification rates and measured A_T_. Shading represents the 95% confidence interval of the linear regression.

There was a positive linear correlation between total calcification and A_T_ (linear model: *p* < 0.00001, R^2^ = 0.44; Fig. 2B) with no genotype effect (linear mixed-effect model: *p* = 0.31). Post-hoc estimated marginal means analysis of treatment groups using Tukey’s HSD revealed that calcification rate increased between the ambient treatment and all groups receiving alkaline solution (*p* < 0.001 for all comparisons), but not between the + 1200, + 1850, and + 2000 groups (Fig. 2A). On average, the growth response plateaus at relatively modest enrichment (1.5 to 1.85 times ambient seawater A_T_), with calcification rates increasing by up to 125% in the highest treatment compared to corals receiving ambient seawater: 0.37 ± 0.05 (mean ± s.e.m.) versus 0.83 ± 0.06 mg cm^−2^ day^−1^.

##### Linear extension

**Fig. 3:**
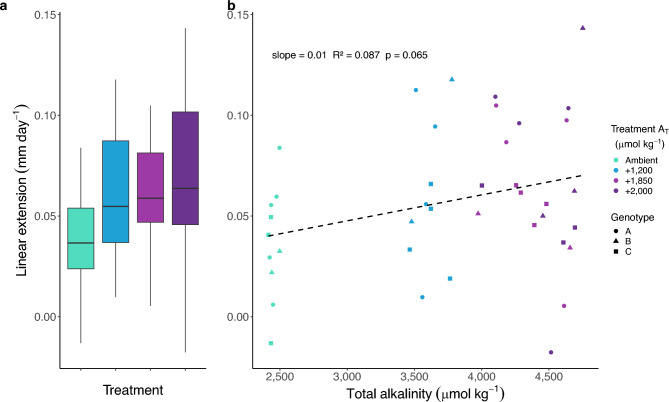
Effect of alkalinity enrichment on *A. cervicornis* linear extension rates (LE). (**A**) Linear extension rates (median ± IQR; n = 10 per treatment). No significant difference between treatment groups (Tukey’s HSD p > 0.05). (**B**) Linear regression between linear extension and measured A_T_**.** Dashed line shows non-significant trend (linear model: p = 0.065).

When calculating LE rates across all timepoints (33 days of growth), linear regression analysis showed no statistically significant correlation with A_T_ (Fig. 3B, linear model: *p* = 0.065, R^2^ = 0.087). Between discrete treatments (Fig. 3A), LE rates of corals in the highest treatment were different from the controls (linear model: *p* = 0.048), but a post hoc estimated marginal means analysis using Tukey’s HSD multiple comparison adjustment reported no significant differences between treatments (all pairwise comparisons: *p* > 0.05). On average, LE rates were 89% higher in the highest alkalinity treatment compared to ambient: 0.04 ± 0.01 (mean ± s.e.m.) versus 0.07 ± 0.01 mm day^−1^. There was no significant genotypic effect (linear mixed-effects model: *p* = 0.60).

#### Reduced calcification and linear extension over time

**Fig. 4:**
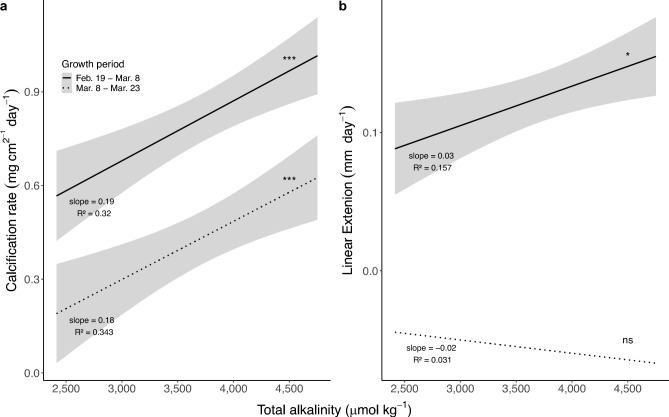
Comparison of growth rates between the first and second half of the experiment (February 19–March 8 versus March 8–March 29). (**A**) Total calcification. (**B**) Linear extension. Significance of the relationship between alkalinity and growth is indicated by asterisks: ***p < 0.001, *p < 0.05, ns = not significant. Post hoc comparisons among treatments were performed using Tukey’s HSD test. Shading represents the 95% confidence interval of the linear regression.

There was a marked decline in coral growth across all treatments during the latter half of the experiment (March 8 to March 23). While total calcification rates decreased for all corals, the treatment effect was retained in the final weeks, and corals continued to show a net gain in mass (Fig. 4A). LE, however, was effectively halted, with rates approaching zero for all corals regardless of treatment (Fig. 4B). During the first half of the experiment, LE was significantly correlated with A_T_ (linear model: *p* = 0.01, R^2^ = 0.157), with mean LE rates increasing by up to 98% in the highest treatment as compared to corals receiving ambient seawater: 0.078 ± 0.01 (mean ± s.e.m.) versus 0.155 ± 0.02 mm day^–1^.

#### Photosynthetic efficiency

Photochemical health was measured once at the final time point. Mean *F*_*v*_*/F*_*m*_ values (0.6 ± 0.007 s.e.m.) did not differ significantly between treatments or genotypes (ANOVA, *p* > 0.05).

## Discussion

### Growth enhancement

This study is the first to establish a significant relationship between enriched alkalinity, total calcification and linear extension in *A. cervicornis*. Calcification rates more than doubled, and linear extension increased by up to 98% during the first half of the experiment, with no obvious impact to coral health based on the limited parameters measured (i.e., survivorship and *F*_*v*_*/F*_*m*_). Corals across all treatments maintained pigmentation through the experiment, and there were no significant differences in photochemical efficiency between the ambient and the enriched conditions, suggesting that elevated alkalinity did not negatively impact coral health. Calcification was increased by 125% and linear extension by 98% (in the first two weeks) at ~ 1.85 times the ambient A_T_ (~ 4600 µmol kg^−1^)_._

Our findings align well with those of Marubini and Thake^33,37^, who observed a doubling of calcification and a 55% increase in linear extension in *Porites porites* following the addition of 2 mM HCO_3_^–^ to ambient seawater. Notably, our study demonstrated an even more pronounced growth response with approximately half the HCO_3_^–^ addition (~ 1 mM), indicating a stronger sensitivity to alkalinity enrichment. This enhanced response is also consistent with the genus-level patterns described by Herfort et al.^34^, in which Pacific *Acropora* sp. exhibited greater responsiveness than Caribbean *P. porites*, likely due to interspecific differences in energy allocation strategies^51,52^. However, direct comparisons between our measurements and the calcification rates reported by Herfort et al.^34^ are challenging due to methodological differences—namely, long-term buoyant weight tracking (33 days) versus short-term alkalinity anomaly technique (single 8-h incubations with rates normalized to chlorophyll concentration)^53^.

It is difficult to compare our rates to those of corals reared in offshore nurseries, as growth is typically tracked using a colony-level growth metric which measures the cumulative, or total linear extension, of all apical tips. In contrast, our measurements focused on single-branch LE and calcification, which may not fully capture total colony growth potential. More directly relevant are rates reported in other ex-situ experiments, including LE of ~ 0.14 mm day^–1^ at 26 °C under ambient conditions^43^. Our highest alkalinity treatments produced comparable rates, suggesting that conditions in the experimental beakers (e.g., feeding regime, light intensity, water flow, or chamber volume) were not fully optimized for long-term *A. cervicornis* growth. This interpretation is further supported by the decline in calcification and LE observed over time in both controls and treated corals (Fig. 4), indicating system-wide constraints on growth independent of alkalinity treatment.

Our results suggest that the relationship between LE and A_T_ is less robust than that for total calcification. While both calcification and LE doubled or nearly doubled during the first half of the experiment, the loss of a treatment effect on LE in the latter half indicates that other environmental factors may strongly influence this process. The primary drivers of densification versus linear extension remain unclear, as both are known to be plastic in many coral species^54,55^. Investment in one or the other likely depends on the interaction of many environmental variables, such as water flow, light exposure, and nutrient levels–in addition to the carbonate chemistry of seawater–with strong genotypic and species variation^42,56,57^. Long-term growth studies in the Great Barrier Reef showing decadal declines in LE hypothesize changes in temperature and Ω_Arg_ as the causative agents^58^, but the literature remains inconclusive. Similar investigations in the Caribbean could not conclusively connect slowing LE rates to a decline in pH^59^, and some species have demonstrated the ability to maintain constant LE despite reduced skeletal mineralization potential under low pH conditions by increasing their skeletal porosity^45^.

While LE may not always directly respond to water chemistry, coral growth rates are highly variable^60,61^, and our results suggest that elevating alkalinity can enhance LE under specific conditions. This is supported by Schutter et al.^31^ who found that coral larvae exposed to both elevated A_T_ and a heterotrophic feeding regimen saw increased survivorship and enhanced skeletal development, possibly reflecting an improved capacity for HCO_3_^–^ utilization. It has also been suggested that the nutritional status of a coral may impact its response to ocean acidification, with nutritionally replete corals able to counteract the negative impacts of *p*CO_2_ on calcification^62–64^. Future work should investigate how to best optimize conditions to encourage LE under elevated alkalinity (e.g., considerations of flow, temperature, nutritional status, and light exposure), as well as explore any changes in skeletal density, porosity, or structure of the skeletal matrix. Additionally, genotypic sensitivity may emerge under longer exposure to alkalinity enrichment, and future studies should continue to evaluate this, particularly given the importance of maintaining and enhancing genetic diversity for coral restoration.

### Challenges with alkalinity enrichment

Within our discrete treatments, calcification and linear extension rates became more variable with increasing A_T_, and no statistical differences in rates were observed among the three alkalinity-supplemented treatments (Figs. 2A, 3A). This may suggest a non-linear relationship between growth and alkalinity or a physiological saturation point beyond which the treatment effect is lost. Additionally, the average difference in A_T_ between beakers with and without corals was less pronounced in the highest treatments (+ 1850 and + 000; Table 1, Fig. 1B), suggesting that ion uptake may have plateaued, consistent with physiological saturation. However, because the two highest alkalinity treatments were not statistically distinct from each other (Fig. 1A), we cannot rule out the possibility that the observed growth plateau reflects increased variability in A_T_ or Ω_Arg_ in the higher treatments, rather than a true biological threshold.

Herfort et al.^34^, for example, observed no signs of growth saturation at an alkalinity of 8000 μM. We were not able to reach targets this high during a pilot trial without significant abiotic precipitation of calcium carbonate in the beakers, and so opted for lower maximum alkalinity treatments. The system’s inability to reach our initial upper target of + 3000 µmol kg^−1^ A_T_ above ambient seawater (~ 5500 µmol kg^−1^ A_T_) may reflect limitations of the enrichment method (i.e., NaHCO_3_ and Na_2_CO_3_), challenges with maintaining stable carbonate chemistry manipulations in small water volumes (600 mL), or a practical upper limit to long-term alkalinity supplementation.

It is also possible that our two highest enriched treatments were different from each other, but water sample storage post-collection impacted the quality of the measurements. While we did remove from analysis any bottle samples with obvious precipitation of calcium carbonate, it is possible that precipitation occurred which was not visually apparent^65^. Because carbonate system parameters are calculated from measured A_T_ and dissolved inorganic carbon (DIC), small uncertainties or undetected precipitation at elevated A_T_ can propagate non-linearly into derived Ω_Arg_ and pCO₂ values, increasing uncertainty in calculated carbonate speciation among the highest treatments and limiting fine-scale interpretation of differences between them. In the future, the pH of any samples with expected high Ω or A_T_ should be lowered by briefly bubbling pure CO_2_ gas at the time of collection to prevent precipitation during storage, as suggested by Schulz et al.^66^. Similarly, precipitation in DIC samples may be avoided without impacting the integrity of the measurement by adding hydrochloric acid. Measuring pH at the time of water sample collection would provide a way to evaluate whether the calculated carbonate chemistry parameters of the beakers using the measured DIC and A_T_ accurately reflect the conditions corals are experiencing in the beakers.

### Implications for coral restoration

While land-based facilities offer increased climate resilience, concerns remain about scalability and the possibility of depressed growth rates compared to corals grown in offshore environments. Nevertheless, rearing corals on land, even temporarily, may become more common as we contend with rising ocean temperatures. While we did not observe sustained LE in this experiment, the continuous treatment effect on total calcification suggests that corals did sustain enhanced lateral thickening or densification, both of which would indicate the capacity to enhance restoration potential either by producing stronger skeletons or increasing the production of coral fragments. These findings are also relevant to considerations of ecological safety under OAE. The enrichment levels achieved here fall within the range suggested for OAE biological research, which recommend testing broad alkalinity gradients of up to several thousand μmol kg^−1^ above ambient to identify ecological thresholds or potential negative organismal responses^67^. Our use of bicarbonate and carbonate salts to elevate alkalinity mimics realistic carbonate-chemistry perturbations under OAE scenarios and produces carbonate-system endpoints that are analogous to multiple proposed OAE feedstocks like olivine and quicklime^67^.

Additionally, the effectiveness of alkalinity manipulation should be investigated for the sexual propagation of coral larvae. Increasing calcification during the larval phase could reduce the time spent in vulnerable early life stages, potentially minimizing juvenile mortality^47^, shortening generation times^48,68,69^, and reducing nursery residence time prior to outplanting^16^.

Both commercial and hobby aquarists commonly manipulate the alkalinity of saltwater tanks, generally identifying the ideal range for carbonate alkalinity as between 3400 and 4100 μmol kg^−1^, or 1.7-times the average measured on natural reefs. We saw no significant difference in calcification or LE between the three treatments receiving alkalinity supplementation, suggesting similar results may be achieved in as low as 1.5-times the ambient seawater alkalinity. While a range of commercial products are available and additional mineral supplementation (e.g., calcium and magnesium) must be considered when manipulating alkalinity in closed-circulation systems, this study demonstrates that in flow-through systems, a simple solution of baking soda and soda ash can more than double calcification, supporting alkalinity enrichment as a scalable and accessible strategy for improving restoration efficiency.

## Methods

### Coral collection and acclimation

Fragments of three presumed distinct *A. cervicornis* genotypes (confirmed via single nucleotide polymorphism, Supplementary Table S1) were collected from the University of Miami’s offshore nursery (25.767451, − 80.145704) on January 11, 2024. Nubbins in the 3–6 cm range with one apical tip per fragment were transported to CIMAS/AOML’s Experimental Reef Lab at the University of Miami Rosenstiel School and transferred to a holding tank set to match the in-situ temperature at the time of collection (23 °C). Corals were allowed to recover and acclimate to the tank conditions for one week before ramping (0.5 °C day^−1^) up to the experimental temperature of 27 °C, which they were held at for an additional 2 weeks. Corals were broadcast-fed 150 mL of ReefRoids (Polyplab) daily at 5:00 pm (3 g mixed in 2 L of distilled water) throughout ramping and acclimation. At the end of the acclimation period, corals which had lost apical tips were excluded from the experiment, yielding a total of 40 corals for investigation (n = 17, 9, and 14 from genotypes Coopers, Marker-9, and Sunny Isle’s-E. Herein referred to as genotypes “A”, “B”, and “C”, respectively).

### Experimental design

The forty coral fragments were randomly distributed by genotype across four treatments (Supplementary Table S2). Each coral was placed into a 600 mL beaker equipped with a stir bar to ensure adequate water flow and gas exchange inside the vessel. Coral fragments were suspended from acrylic beams seated across the top of the beakers using a looped monofilament to promote LE, as is common practice in restoration nurseries. Two extra beakers per treatment were left empty to assess how carbonate chemistry was altered in the absence of corals. Beakers were then divided between four glass tanks (12 beakers per tank) and semi-submerged in a water bath for temperature control. Each beaker was treated as an independent experimental unit. All beakers (including those without corals) were dosed five times a week at 17:00 with ReefRoids, yielding a final concentration of ~ 15 mg L^−1^ per feeding event, consistent with common land-based aquaculture practices used in coral nurseries.

Water turnover in each beaker, along with treatment administration, was facilitated by the Sequential Treatment Application Robot (STAR) system^50^. The system consists of two robotic arms (xArm 6, Ufactory) fitted with custom-built end effectors equipped with two pipette tips. Tubing connects the tips to a dosing box, with one line terminating at a brushless peristaltic pump and the other at a 2.5 mL syringe pump. Each robotic arm served two tanks (24 beakers each), moving sequentially from beaker to beaker, dosing ambient seawater via the peristaltic pump and a concentrated alkaline solution via the syringe. Each robot completed a full dosing cycle every 21.5 min, resulting in approximately 4.5 water changes in each beaker day^−1^.

Ambient seawater for the beakers was sourced from Bear Cut in Biscayne Bay, passed through UV sterilization, and then sequentially filtered through 25, 5, and 1 μm filter socks. The filtered water was then routed to two independent glass tank reservoirs in circulation with 75 L sumps at approximately 400 mL min^–1^, resulting in roughly 3.8 water changes per day. Each reservoir supplied one robot. Effluent from the dosed beakers replenished the surrounding water bath, which was in circulation with the experimental tank’s flow-through sump and continuously drained via overflow. The temperature of the water baths was logged every five minutes using a high-accuracy Resistance Temperature Detector sensor and maintained at 27 °C using a 300 W aquarium heater and a titanium chiller coil housed in the sump as described in Enochs et al.^70^. Each tank is fitted with a high-intensity LED light array programmed to simulate natural diel light cycles, with peak photosynthetically active radiation (200 µmol m^−2^ s^−1^) reflecting mid-range irradiance within the typical depth distribution (5–20 m) of *A. cervicornis.* Lights followed a scheduled 12-h regime, ramping up from zero intensity between 06:00 and 09:00, maintaining maximum intensity from 09:00 to 15:00, and ramping down from 15:00 to 18:00.

### Enriched alkalinity treatments

The four treatments included an ambient seawater control, with A_T_ typically ~ 2500 µmol kg^−1^, and three elevated treatments targeting + 1500, + 3000, and + 4500 µmol kg^−1^ above ambient A_T_. However, the treatment levels are renamed to reflect the actual measured A_T_ achieved in the experiment: ambient, + 1200, + 1850, and + 2000, where values indicate µmol kg⁻^1^ A_T_ above ambient. Food-grade NaHCO_3_ (baking soda) and Na_2_CO_3_ (soda ash; produced by baking NaHCO_3_ at ~ 204 °C for one hour) were mixed with reverse-osmosis water at a 1.26:1 weight ratio to create a concentrated alkaline solution (A_T_ ~ 220,000 µmol kg^−1^). We chose this ratio to maintain a constant *p*CO_2_ of ~ 400 µatm in the beakers across a range of alkalinity, allowing pH to vary. For reference, see Supplementary Fig. S1 online for a property-property plot demonstrating how varying proportions of HCO_3_^–^ and CO_3_^2−^ can be used to achieve this target. Treatments were administered and maintained in beakers by dosing 40 mL of ambient seawater and varying volumes of the concentrated alkaline solution every 21.5 min (0.22, 0.44, or 0.66 mL to the + 1200, + 1850, and + 2000 treatments, respectively). Corals were placed in their beakers with ambient seawater on February 4 and gradually acclimated to their treatments by incrementally increasing the alkaline solution dose by 0.22 mL per day to minimize stress. All treatments reached full dosing volume by the end of day February 6, and the regimen was maintained for seven weeks.

### Carbonate chemistry analysis

To characterize carbonate chemistry, paired seawater samples were collected once a week between 10:00 and 13:00 for A_T_ and DIC analysis (200 mL and 125 mL, respectively). A second independent A_T_ sample was collected later in the week to monitor the stability of the treatments over time. The A_T_ samples were filtered through a 0.45 µm combusted glass fiber filter and preserved in screw-top borosilicate glass bottles using 150 µL of 6.5% mercuric chloride (HgCl_2_). Samples for the determination of DIC were collected in borosilicate bottles with ground glass stoppers, preserved with 100 µL of HgCl_2,_ and sealed with Apiezon grease. The A_T_ samples were analyzed in 50 g duplicates using a potentiometric titrator (855 Robotic Titrosampler, Metrohm) equipped with an 800 Dosino pump and controlled using Tiamo software. The DIC samples were analyzed using an AS-C3 (Apollo SciTech), also in 50 g duplicates. If the replicates for either parameter differed by more than 5 μmol, a third replicate was analyzed. Samples were excluded from further analysis if the third replicate did not fall within 5 μmol of at least one of the previous measurements. The mean of the sample replicates within 5 μmol was used for further calculations. Both DIC and A_T_ samples were calibrated with certified reference materials following Dickson et al.^71^. All samples were analyzed within one month of collection.

Beginning in week four of the experiment, precipitation of calcium carbonate was observed in some of the sampling bottles post-collection for the + 1850 and + 2000 treatments, leading to higher variability in the measured A_T_ and DIC. Samples with visible precipitation post-collection were also excluded from further analysis.

The remaining weekly paired A_T_ and DIC measurements were used to generate additional carbonate chemistry parameters (e.g., pH and Ω_Arg_) using the *seacarb* package^72^ in the R software environment (v4.3.2; R Core Team 2023). Several samples produced abnormally high *p*CO_2_ values in *seacarb*, but were not flagged as having visible precipitation in the bottles. It is possible that there was precipitation in these samples, but it was not caught or possibly not visible to the naked eye. Based on this, another six samples with *p*CO_2_ > 2,000 µatm were excluded from further analysis.

In total, 141 out of the original 859 samples were excluded, leaving 642 remaining for data analysis (393 A_T_ and 249 DIC). See Supplementary Table S3 for a detailed breakdown of samples collected, excluded, and retained by treatment. The treatment level means and standard error of the means (± s.e.m.) for carbonate chemistry parameters were calculated using all valid bottle samples, including both coral-containing beakers and those without corals. For analyses of the relationship between alkalinity and coral growth, the mean A_T_ (± s.e.m.) was calculated for each coral based on the two weekly A_T_ measurements from its beaker. As a result, the dataset used for alkalinity-based growth analyses included more discrete bottle samples (n = 326) than the dataset used to generate carbonate chemistry parameters (n = 249).

### Coral growth

Initial weights and lengths of fragments (specifics described below) were collected on February 19th after all corals had acclimated to beaker and treatment conditions. Additional buoyant weight and LE measurements were collected on March 8 and March 23. Six of the corals experienced partial tissue loss in the second half of the experiment and were removed from the treatments. For those corals, growth was assumed to have stopped the day they started showing signs of decline and that day was used as the endpoint to calculate their growth rates. For all other corals, growth rates represent 33 days of growth. The partial mortality did not appear to be associated with treatment (two out of six were controls, three were receiving the + 1,200 treatment, and one was in the + 2000 group). Five of the six were of the same genotype (A).

#### Surface area

Fragments were 3D-scanned prior to the experiment using an assembly consisting of two Basler A2040 cameras equipped with Fujinon lenses and connected to a Benq projector. 3D models were rendered using FlexScan3D (Polyga, version 3.3), and surface area measurements (1.2% coefficient of variance) were extracted from the models using Leios2 software (EG Solutions, R10 Rev.0 Build 64).

#### Total calcification

Total calcification was quantified with the buoyant weight technique^73^ using a calibrated analytical balance (0.0001 g precision, Ohaus). Corals were placed in a stainless-steel wire basket submerged in saltwater, suspended from the scale by a tungsten microfilament (0.05 mm). Data were converted to dry skeletal weight following the method of Jokiel et al.^74^, using salinity and temperature measurements recorded with an EcoSense EC300A (YSI). Calcification rates (mg cm^-2^ day^-1^) were calculated as the changes in dry skeletal weight divided by the number of days between measurements and standardized to the initial surface area of each coral.

#### Linear extension

LE rates (mm day^−1^) were calculated by analyzing photographs collected at each time point with ImageJ (V.1.54). Pictures were taken using a PowerShot G1X (Canon) affixed to a custom-built stage and mount to ensure the corals were positioned the same distance from the camera at each time point. The growth axis of the coral was traced with a segmented line tool from base to tip to determine total length. Previous photos were referenced to ensure corals were oriented in the same position and that the growth axis was traced along the same path across time points. To account for slight changes in camera position or angle between time points, standards were used to generate an offset (Supplementary Fig. S2). The mean length of the standard from the first time point (February 19) was used as the baseline because it had the least amount of variability. Offsets were generated for the other two time points and applied to individual coral lengths (March 8, + 0.873 mm; March 28, + 0.383 mm). The correction method had a standard deviation of ± 0.40 mm and a standard error of ± 0.12 mm (relative standard error of 0.5%), based on repeated measurements of the photographic scale standards across all time points. All LE analyses were based on offset-corrected coral lengths.

### Photophysiology

Photosynthetic efficiency (*F*_*v*_*/F*_*m*_) of the coral’s algal symbionts was evaluated by measuring the maximum quantum yield of photosystem II fluorescence using a Maxi Imaging-PAM fluorometer (Walz) on day 33 following 30 min of dark adaptation. Imaging-PAM instrument settings as follows: measuring light (ML) intensity = 1, ML frequency = 1, saturation pulse (SP) intensity = 7, SP width = 240 ms (Width x60ms = 4), gain = 4, and damping = 2. The ratio of variable fluorescence (*F*_*v*_) to maximum fluorescence (*F*_*m*_) was calculated as a proxy for the algal community’s photosynthetic health.

### Statistics

Statistical analyses and figures were generated using the R software environment (version 4.3.2; R Core Team 2023). The relationship between total calcification and LE rates with A_T_ was investigated using linear mixed-effect models in the *lme4* package^75^ (version 1.1–31). Models were generated using A_T_ both as a continuous variable and grouped into discrete treatments, with growth parameter (calcification or LE) and A_T_ as fixed effects. Genotype was initially evaluated as a fixed effect but was not significant. In the final mixed-effect models, both genotype and tank were treated as random effects. Analysis of variance (ANOVA) models assessed statistical differences between treatments. Tukey’s HSD pairwise comparisons for significant factors were performed using *emmeans* version 1.1.3 with an alpha of 0.05^76^. All figures were generated with ggplot2^77^.

## Supplementary Information

Supplementary Information.

## Data Availability

Data and code produced as part of this study are publicly available in the Zenodo repository at: 10.5281/zenodo.15784937.
