# Supplementary material for: Alkalinity enrichment stimulates calcification and linear extension in Acropora cervicornis
Source: Sci Rep. 2026 Mar 22;16:14512. doi: 10.1038/s41598-026-44817-6 (PMC13150020; doi:10.1038/s41598-026-44817-6)
Supplement: Supplementary file 1 — Supplementary Information. [file 41598_2026_44817_MOESM1_ESM.docx]

**Supplementary Information**

**Title:** Alkalinity enrichment stimulates calcification and linear extension in *Acropora cervicornis*

Kenzie M. Cooke^1,3*^, Ana M. Palacio-Castro^1^, Albert Boyd^1^, Nash Soderberg^1^, Patrick M. Kiel^1,3^, Ashley Stevens^1^, Chris Langdon^3^, Ian C. Enochs^2^

^1^ Cooperative Institute for Marine and Atmospheric Studies, Miami, FL, USA

^2^ Atlantic Oceanographic and Meteorological Laboratory, Miami, FL, USA

^3^ Rosenstiel School of Marine, Atmospheric, and Earth Science, University of Miami,

Miami, FL, USA

*Corresponding author: Kenzie McKenna Cooke (kmc390@miami.edu)

**Supplementary Table S1**. Genotype identifiers for genets used in this study, pulled from Acropora Cervicornis Data Coordination Hub[1]. <https://coral.aoml.noaa.gov/AcDC/>.

| **Genotype identifier** | | | | |
| --- | --- | --- | --- | --- |
| *General ID* | *Local name* | *STAGdb ID* | *Microsatellite ID* | *CSR Accession* |
| A | Cooper’s | HG0949 | C1350 | 46f36817-7c86-2f92-1ca5-cd9bf45ce5fa |
| B | Marker-9 | HG0949 | C1355 | be206fae-10b5-2bbc-a909-3394fa360315 |
| C | Sunny Isle’s E | N/A | N/A | ccbfc660-891d-bfda-23de-f68bf0fc66e2 |

**Supplementary Table S2.** Summary of experimental replicates across treatments.

|  |  | **Total alkalinity treatment** | | | |
| --- | --- | --- | --- | --- | --- |
|  |  | Ambient | +1,200 | +1,850 | +2,000 |
| **Genotype** | **n** | **Coral replicates** | | | |
| A | 17 | 5 | 4 | 4 | 4 |
| B | 9 | 2 | 2 | 2 | 3 |
| C | 14 | 3 | 4 | 4 | 3 |
| ***Total Coral*** | **40** | **10** | **10** | **10** | **10** |
|  |  | **Empty beaker replicates** | | | |
| ***Total Blank*** |  | **2** | **2** | **2** | **2** |

**Supplementary Figure S1.** Property–property plots illustrating how a constant *p*CO₂ of ~400 µatm can be maintained by adjusting total alkalinity and dissolved inorganic carbon in a specific ratio. Dots represent the four target treatment alkalinities and black lines are *p*CO₂ isocontours (µatm). **A** (top) and **B** (bottom) depict the effects on *p*CO_2_ when increasing alkalinity using sodium carbonate only and sodium bicarbonate only, respectively. When used in combination at the appropriate ratio, these two compounds can elevate alkalinity without altering *p*CO₂. **C** The solid circles represent ideal theoretical A_T_:DIC combinations that fall directly along the 400 µatm isocontour for the selected alkalinity targets. The stars depict the “actual” treatment parameters achieved during the experiment.

**Supplementary Table S3.** Summary of water samples collected and used for carbonate chemistry analysis. Each beaker was sampled twice per week for A_T_ and once per week for DIC. All bottle samples were analyzed but were excluded from further data analysis if they developed visible abiotic precipitation of calcium carbonate during storage and/or if the paired A_T_/DIC samples produced a *p*CO_2_ value >2000 μatm when run through *seacarb*. “Remaining” reflects the total number of bottle samples that were left and therefore used in further data analyses. Seacarb *n* values refer to the number of A_T_/DIC pairs used to compute the full carbonate system parameters by treatment. “A_T_ (for regression)” refers to the number of samples used to calculate the average A_T_ per beaker/per coral and used in regression analysis with coral growth.

|  |  |  | **Total alkalinity treatment** | | | | |
| --- | --- | --- | --- | --- | --- | --- | --- |
|  |  | **Beaker Content** | Ambient | +1,200 | +1,850 | +2,000 | ***Total n*** |
| **Collected** | *A_T_* | All | 132 | 132 | 132 | 130 | 526 |
|  | *DIC* | All | 84 | 84 | 84 | 81 | 333 |
|  | ***Total n*** |  | 216 | 216 | 216 | 211 | **859** |
| **Removed** | *∆ > 5 μmol* | All | 4 | 17 | 32 | 49 | 102 |
|  | *Precipitation* | All | 0 | 0 | 16 | 17 | 33 |
|  | *High pCO2*  *(>2000 μatm)* | All | 0 | 0 | 1 | 5 | 6 |
|  | ***Total n*** |  | 4 | 17 | 49 | 71 | **141** |
| **Remaining** | *A_T_* | All | 128 | 115 | 87 | 63 | 393 |
|  | *DIC* | All | 82 | 72 | 55 | 40 | 249 |
|  | ***Total n*** |  | 210 | 187 | 142 | 103 | **642** |
| ***n used in Data Analysis*** | *Pairs of A_T_/DIC (for Seacarb)* | Coral | 68 | 61 | 44 | 33 | 206 |
|  |  | Empty | 14 | 11 | 11 | 7 | 43 |
|  | ***Total n*** |  | 82 | 72 | 55 | 40 | **249** |
|  | *A_T_ (for regression)* | Coral | 106 | 97 | 71 | 52 | 326 |
|  |  | Empty | 22 | 18 | 16 | 11 | 67 |
|  | ***Total n*** |  | 128 | 115 | 87 | 63 | **393** |


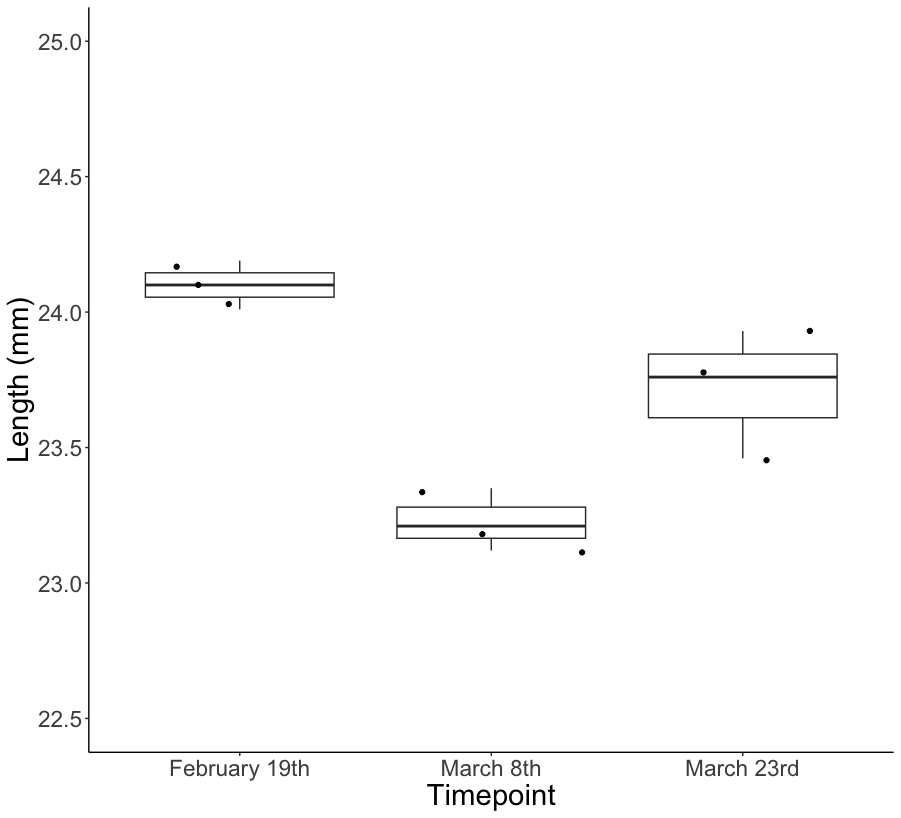


**Supplementary Figure S2.**  Length of a standard measured across time points for linear extension analysis. An offset was generated using the mean length of the standards from T1 and applied to T2 and T3 measurements.

**References**

[1] Kiel, P.M., Formel, N., Jankulak, M., *et al.* Acropora cervicornis data coordination hub, an open access database for evaluating genet performance. *Bull. Mar. Sci.* **99**, 119–136 (2023)
